# Stratified cardiovascular risk in patients with metabolic dysfunction-associated steatotic liver disease (MASLD): impact of varying metabolic risk factor burden

**DOI:** 10.1186/s40001-025-03151-9

**Published:** 2025-09-23

**Authors:** Maha Elsabaawy, Mohamed Torkey, Mai Magdy, Madiha Naguib

**Affiliations:** 1 Department of Hepatology and Gastroenterology, National Liver Institute, Menoufia University, Shebeen Elkoom, Menoufia Egypt; 2 Department of Cardiology, Shebeen Elkoom Teaching Hospital, Shebeen Elkoom, Menoufia Egypt

**Keywords:** Non-alcoholic steatotic liver disease, Cardiovascular diseases, Risk assessment

## Abstract

**Background:**

Metabolic Dysfunction-Associated Steatotic Liver Disease (MASLD) is a spectrum of hepatic disorders that are closely linked to metabolic risk factors. This study aimed to investigate the stratified cardiovascular risk in patients with MASLD based on varying metabolic risk burdens.

**Methods:**

In a cross-sectional analysis, 357 patients with MASLD were classified into three subtypes based on the number of metabolic conditions used for diagnosis: subtype 1 with one metabolic risk factor (n = 110), subtype 2 with two (n = 161), and subtype 3 with three risk factors (n = 86). Clinical parameters, liver enzymes, lipid profiles, and 10-year risk of Atherosclerotic Cardiovascular Disease (ASCVD) were compared among subtypes.

**Results:**

The prevalence of diabetes and hypertension increased significantly across subtypes, with subtype 3 showing the highest burden of cardiovascular risk factors (100% diabetes, 43% hypertension). Cardiovascular risk was elevated in subtype 3 (mean ASCVD 0.112 ± 0.108, p < 0.001). Multivariate analysis identified subtype 3 (OR = 7.812, 95% CI 1.980–30.819, p = 0.003), men sex (OR = 33.549, 95% CI 11.814–95.269, p < 0.001), and age (OR = 1.171, 95% CI 1.116–1.229, p < 0.001) as independent predictors of intermediate to high cardiovascular risk.

**Conclusion:**

The metabolic burden in patients with MASLD significantly stratified cardiovascular risk. Patients with the highest metabolic dysfunction are at increased risk of cardiovascular events, underscoring the need for targeted cardiovascular prevention strategies.

## Introduction

In 2023, an international Delphi consensus panel redefined non-alcoholic fatty liver disease (NAFLD) as metabolic dysfunction-associated steatotic liver disease (MASLD), reflecting its metabolic origins and simplifying diagnosis. Unlike NAFLD, which required exclusion of other liver diseases, MASLD is diagnosed by the presence of hepatic steatosis plus at least one of five cardiometabolic risk factors (e.g., obesity, type 2 diabetes [T2DM], hypertension, dyslipidemia, or insulin resistance), regardless of alcohol use or other liver disease etiologies [1]. This shift acknowledges MASLD as the hepatic manifestation of metabolic syndrome and aligns with its systemic metabolic drivers [2].

MASLD is now the most prevalent chronic liver disease globally, affecting 32.8% of adults, with rates exceeding 50% in populations with obesity or T2DM [3]. Its progressive subtype, metabolic dysfunction-associated steatohepatitis (MASH), accounts for 20–30% of cases and drives rising morbidity from cirrhosis, hepatocellular carcinoma (HCC), and liver-related mortality [4]. Notably, MASLD is projected to become the leading indication for liver transplantation in the U.S. by 2030, surpassing hepatitis C and alcohol-associated liver disease [5].

Critically, MASLD independently increases cardiovascular disease (CVD) risk—the primary cause of death in this population. Shared pathways (e.g., insulin resistance, chronic inflammation, endothelial dysfunction, and atherogenic dyslipidemia) underpin the bidirectional MASLD-CVD relationship [6].

However, it remains unclear whether the number or type of metabolic risk factors influences cardiovascular risk in Patients with MASLD remains unclear. This study aims to stratify cardiovascular risk in Patients with MASLD based on the number of metabolic conditions they meet, providing clearer guidance for clinical management and prevention strategies.

## Methods

### Study design and methodology

This cross-sectional observational study was conducted at the National Liver Institute, Menoufia University, Egypt, between January 2023 and January 2024. We consecutively enrolled 357 adult patients meeting the 2023 international consensus criteria for MASLD [1].

### Diagnostic criteria

1. Hepatic Steatosis: Confirmed via abdominal ultrasonography using standardized criteria (≥ 5% hepatocyte fat accumulation) [7].
2. Metabolic Dysfunction: Defined by the presence of ≥ 1 of the following cardiometabolic risk factors [5]:
3. Overweight/Obesity: BMI ≥ 25 kg/m^2^ (Asian-adjusted thresholds applied where appropriate)
4. Type 2 Diabetes Mellitus (T2DM): HbA1c ≥ 6.5% or fasting glucose ≥ 126 mg/dL
5. Metabolic Dysregulation: Any 2 + of:
6. Waist circumference ≥ 94 cm (M)/80 cm (F) (IDF criteria)
7. Blood pressure ≥ 130/85 mmHg or antihypertensive use
8. Fasting triglycerides ≥ 150 mg/dL
9. HDL cholesterol < 40 mg/dL (M)/ < 50 mg/dL (F)
10. Prediabetes (HbA1c 5.7–6.4%)

Patients were excluded if they had a history of significant alcohol intake (> 30 g/day for men, > 20 g/day for women), viral hepatitis (HBV or HCV), autoimmune liver disease, drug-induced liver injury, or other chronic liver diseases.

### Sample size calculation

The sample size was calculated to ensure adequate statistical power to detect differences in the 10-year Atherosclerotic Cardiovascular Disease (ASCVD) risk score (primary outcome) among the three MASLD subtypes using one-way ANOVA. Based on prior studies, we assumed a moderate effect size (Cohen’s f = 0.25) for differences in ASCVD risk scores across subtypes, reflecting clinically meaningful variations in cardiovascular risk [9]. The following parameters were used:

- Power: 80% (β = 0.20)
- Significance level: 5% (α = 0.05, two-sided)
- Effect size: Cohen’s f = 0.25 (moderate effect)
- Number of groups: 3 (Subtypes 1, 2, and 3)
- Expected variability: Standard deviation of ASCVD risk score ≈ 0.10, based on preliminary data from MASLD cohorts [8, 9].

Using these parameters, the required sample size per group was estimated at approximately 52 patients, yielding a total of 156 patients (52 × 3). To account for unequal group sizes (as observed: n = 110, 161, 86), potential missing data (e.g., incomplete laboratory results), and to ensure sufficient power for secondary analyses (e.g., multivariate logistic regression for predictors of intermediate/high ASCVD risk), the sample size increased by 20%. This adjustment resulted in a target enrollment of 312 patients. The final sample of 357 patients was achieved through consecutive enrollment, providing additional robustness to detect smaller effect sizes and accommodate subgroup comparisons. The calculation was performed using G*Power software (version 3.1.9.7).

### Clinical and laboratory assessment

All participants underwent detailed clinical evaluations, including demographic data, anthropometric measurements (weight, height, BMI, waist circumference), blood pressure, and medical history (hypertension, diabetes, medication use). Laboratory tests included liver function tests (ALT, AST, GGT, bilirubin, albumin, INR), fasting glucose, lipid profile (LDL, HDL, triglycerides), and HbA1c. Fasting insulin was also measured to calculate insulin resistance indices.

The following indices were calculated:

- HOMA-IR (Homeostasis Model Assessment of Insulin Resistance) = [fasting insulin (µU/mL) × fasting glucose (mg/dL)] / 405 [10].
- QUICKI (Quantitative Insulin Sensitivity Check Index) = 1 / [log (fasting insulin) + log(fasting glucose)] [10].
- FIB-4 = (Age × AST) / (Platelets × √ALT) [11].
- NFS, APRI, HIS, and FLI were calculated using standard formulas as validated in previous studies [12, 13].

### Cardiovascular risk assessment

Ten-year atherosclerotic cardiovascular disease (ASCVD) risk was estimated using the pooled cohort equations developed by the American College of Cardiology/American Heart Association (ACC/AHA), which incorporate age, sex, total and HDL cholesterol, blood pressure, diabetes status, and smoking history [14]. Participants were categorized as having low (< 5%), borderline (5–7.4%), intermediate (7.5–19.9%), or high (≥ 20%) ASCVD risk.

### Subtyping of MASLD

Patients with MASLD were classified into three subtypes strictly based on the number of metabolic risk factors required for diagnosis:

Subtype 1: Presence of only one diagnostic criterion (either obesity, type 2 diabetes mellitus, or metabolic dysregulation). Clinically, these patients demonstrated a low prevalence of diabetes (2.7%), high prevalence of overweight/obesity (BMI ≥ 25 in 92.7%), and minimal clustering of other metabolic abnormalities.

Subtype 2: Presence of two diagnostic criteria (any combination of the above). This group showed higher prevalence of diabetes (29.2%), nearly universal obesity (98.8%), and a moderate clustering of metabolic abnormalities, reflecting an intermediate cardiovascular risk profile.

Subtype 3: Presence of all three diagnostic criteria simultaneously. All patients had diabetes and obesity, and 100% met criteria for metabolic dysregulation. This group exhibited the highest metabolic burden, with older age (50.8 ± 9.25 years), highest BMI (37.07 ± 6.14), higher prevalence of hypertension (43%), dyslipidemia (LDL 170.7 ± 65.41 mg/dL; TG 191.2 ± 63.9 mg/dL), and the highest ASCVD scores, signifying the greatest cardiovascular risk.

### Statistical analysis

Data was analyzed using SPSS version 20.0 (IBM Corp., Armonk, NY, USA). Continuous variables were expressed as mean ± standard deviation or median (IQR) as appropriate. Categorical variables were summarized as frequencies and percentages. Differences among subtypes were compared using Chi-square or Fisher’s exact tests for categorical variables, and ANOVA or Kruskal–Wallis tests for continuous variables. Multivariate logistic regression was used to identify predictors of intermediate/high ASCVD risk. A two-sided p-value < 0.05 was considered statistically significant.

## Results

**Fig. 1:**
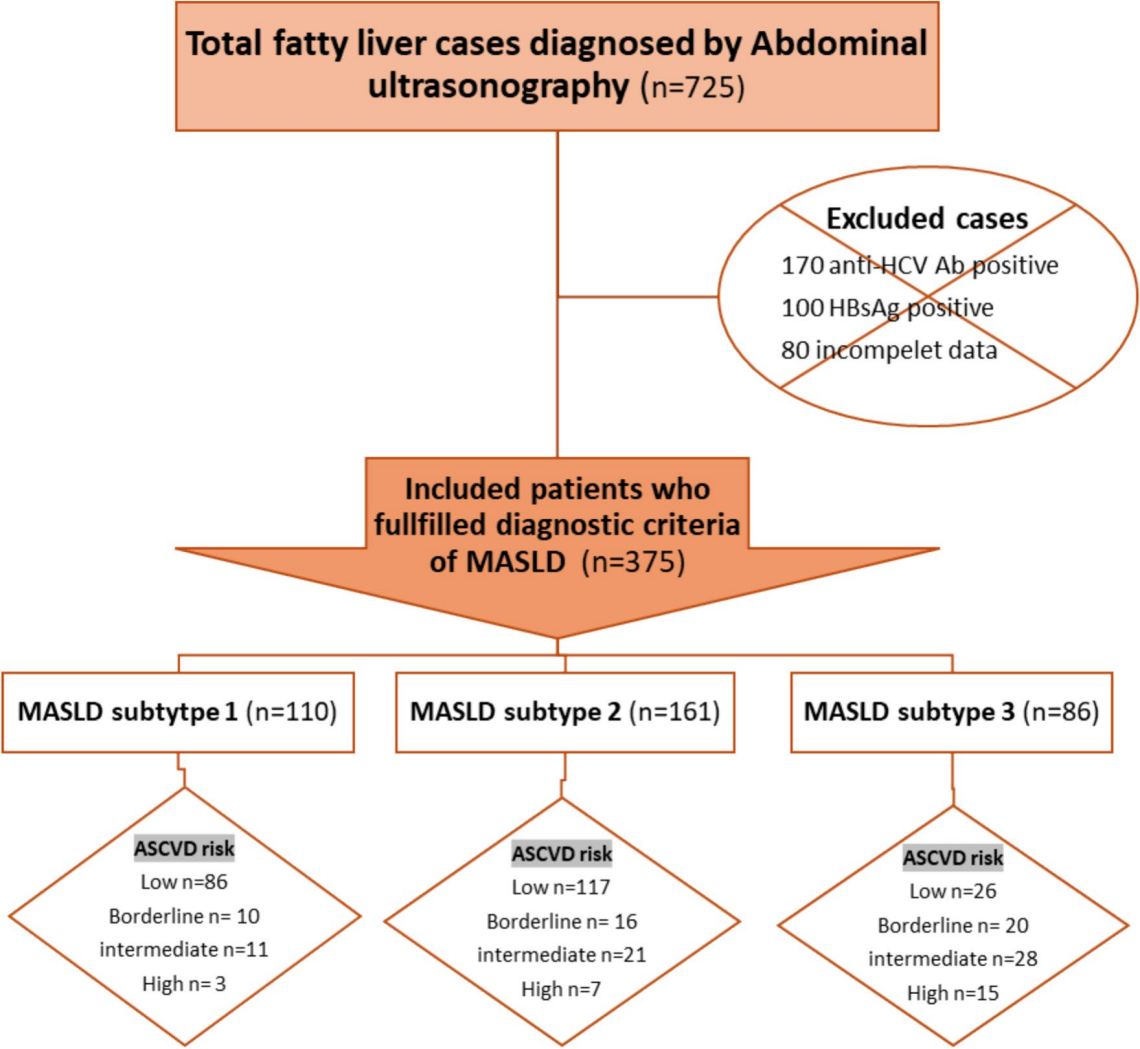
Flow chart of enrolled cases

**Fig. 2:**
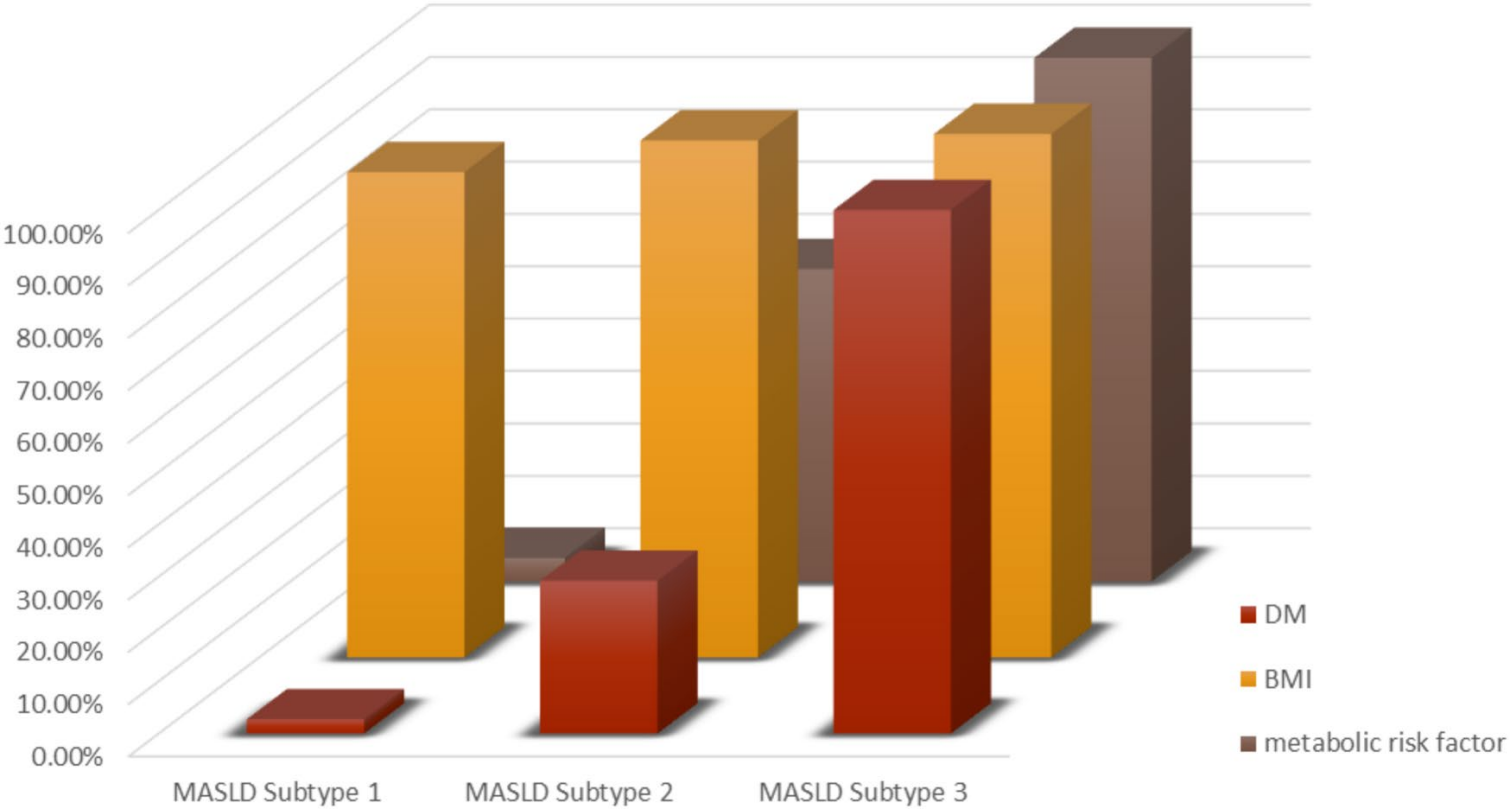
Distributions of the three subtypes according to MASLD diagnostic criteria

A total of 357 patients with MASLD were enrolled and classified into three subtypes based on the number of metabolic risk factors: Subtype 1 (overweight/obese only, n = 110), Subtype 2 (diabetic only, n = 161), and Subtype 3 (diabetic with overweight/obesity and/or ≥ 2 metabolic abnormalities, n = 86) (Fig. 1). The distribution of demographic, clinical, and metabolic characteristics across these subtypes is summarized in Fig. 2.

Patients in Subtype 3 were significantly older (mean age 50.83 ± 9.25 years), with high prevalence of hypertension (43%) than in Subtype 1 (12%) and Subtype 2 (15%; p < 0.001). All patients in Subtype 3 had diabetes and obesity, with 100% exhibiting ≥ 2 metabolic risk factors. Subtype 1 had a low prevalence of diabetes (2.7%) but a high rate of obesity (92.7%), while Subtype 2 showed intermediate characteristics, with 29.2% having diabetes and 98.8% obesity (p < 0.001 for all comparisons) (Table 1).

**Table 1:** Demographic, clinical, and biochemical characteristics of MASLD subtypes

		MAFLD subtypes			Test of Sig	P	Sig. s
		Subtype 1 (n = 110)	Subtype 2 (n = 161)	Subtype 3 (n = 86)			
Sex							
Men		38 (34.5%)	54 (33.5%)	26 (30.2%)			
Women		72 (65.5%)	107 (66.5%)	60 (69.8%)	χ2 = 0.437	0.804	–
Age (years) M ± SD		47.11 ± 11.05	45.78 ± 11.02	50.83 ± 9.25	H = 15.779*	< 0.001*	p1 = 0.262, p2 = 0.007*, p3 < 0.001*
HTN		6 (5.5%)	22 (13.7%)	37 (43.0%)	χ2 = 49.807*	< 0.001*	–
DM		3 (2.7%)	47 (29.2%)	86 (100%)	χ2 = 198.86*	< 0.001*	–
BMI (kg/m^2^) M ± SD		34.21 ± 5.79	33.29 ± 5.89	37.07 ± 6.14	H = 22.4*	< 0.001*	p1 = 0.085, p2 = 0.004*, p3 < 0.001*
WC M ± SD		111.5 ± 11.96	112.8 ± 14.12	116.5 ± 12.10	H = 7.518*	0.023*	p1 = 0.339, p2 = 0.007*, p3 = 0.043*
AST M ± SD		36.44 ± 20.03	35.97 ± 20.42	37.77 ± 21.05	H = 1.231	0.540	–
ALT M ± SD		41.71 ± 20.21	44.10 ± 18.69	38.14 ± 19.87	H = 0.495	0.005*	p1 = 0.072, p2 = 0.160, p3 = 0.001*
GGT M ± SD		43.30 ± 28.82	43.79 ± 34.60	43.36 ± 24.11	H = 2.931	0.781	–
INR M ± SD		1.03 ± 0.06	1.02 ± 0.06	1.01 ± 0.06	H = 2.933	0.231	–
Platelets M ± SD		228.1 ± 59.47	224.9 ± 55.01	233.7 ± 45.04	H = 2.933	0.231	–
TC M ± SD		208.2 ± 57.37	209.1 ± 56.99	256.9 ± 60.27	H = 42.228*	< 0.001*	p_1_ = 0.949, p_2_ < 0.001^*^, p_3_ < 0.001^*^
HDL M ± SD		54.44 ± 18.34	48.62 ± 23.08	48.01 ± 22.93	H = 34.486*	< 0.001*	p1 < 0.001*, p2 < 0.001*, p3 = 0.825
LDL M ± SD		127.7 ± 57.75	128.8 ± 52.65	170.7 ± 65.41	H = 34.412*	< 0.001*	p1 = 0.826, p2 < 0.001*, p3 < 0.001*
TG M ± SD		130.2 ± 53.8	158.5 ± 85.6	191.2 ± 63.9	H = 53.843*	< 0.001*	p1 = 0.006*, p2 < 0.001*, p3 < 0.001*
HbA1C M ± SD		5.33 ± 0.64	5.73 ± 1.12	7.53 ± 1.83	H = 115.829	< 0.001*	p1 = 0.023*, p2 < 0.001*, p3 < 0.001*
HOMA-IR M ± SD		0.35 ± 0.03	0.33 ± 0.03	0.32 ± 0.04	H = 53.333	< 0.001*	p1 < 0.001*, p2 < 0.001*, p3 = 0.009*
ASCVD M ± SD		0.041 ± 0.068	0.047 ± 0.071	0.112 ± 0.108	H = 67.188*	< 0.001*	p1 = 0.389, p2 < 0.001*, p3 < 0.001*
ASCVD interpretations							
Low	86 (78.2%)		10 (9.1%)	11 (10.0%)	χ2 = 60.552*	< 0.001*	–
Borderline	117 (72.7%)		16 (9.9%)	21 (13.0%)			
Intermediate	26 (30.2%)		20 (23.3%)	25 (29.1%)			
High	3 (2.7%)		7 (4.3%)	15 (17.4%)			
APRI M ± SD	0.53 ± 0.33		0.54 ± 0.38	0.52 ± 0.32	H = 0.009	0.996	–
ALBI M ± SD	− 3.75 ± 0.31		−3.70 ± 0.28	−3.63 ± 0.30	H = 6.903*	0.032*	p1 = 0.184 p2 = 0.009* p3 = 0.109
FIB4 M ± SD	1.28 ± 0.84		1.20 ± 0.87	1.40 ± 0.78	H = 11.060*	0.004*	p1 = 0.312 p2 = 0.027* p3 = 0.001*
NFS M ± SD	−1.64 ± 1.28		−1.45 ± 1.41	0.01 ± 1.02	H = 82.148*	< 0.001*	p1 = 0.305 p2 < 0.001* p3 < 0.001*
HIS M ± SD	45.68 ± 7.74		46.51 ± 7.60	49.28 ± 7.86	H = 11.047*	0.004*	p1 = 0.420 p2 = 0.001* p3 = 0.007*
FLI M ± SD	83.25 ± 14.72		82.62 ± 17.26	92.20 ± 8.84	H = 28.408*	< 0.001*	p1 = 0.757 p2 < 0.001* p3 < 0.001*

Values are presented as mean ± SD or n (%). P-values derived from ANOVA for continuous variables and Chi-square test for categorical variables. *HTN* hypertension, *DM* diabetes mellitus, *BMI* body mass index, *AST* aspartate aminotransferase, *ALT* alanine aminotransferase, *GGT* gamma-glutamyl transferase, *INR* international normalized ratio, *HDL* high-density lipoprotein, *LDL* low-density lipoprotein, *TG* triglycerides, *HbA1C* hemoglobin A1c, *WC* waist circumference, *W/H* waist-to-hip ratio, *HOMA-IR* homeostasis model assessment of insulin resistance, *QUIKI* quantitative insulin sensitivity check index, *APRI* AST-to-platelet ratio index, *ALBI* albumin-bilirubin index, *FIB-4* fibrosis-4 score, *HIS* hepatic steatosis index, *FLI* fatty liver index, *NFS* NAFLD fibrosis score, *M* mean, *SD* standard deviation, *OR* odds ratio, *CI* confidence interval, *QR* Inter quartile range, *SD* Standard deviation, *χ*^*2*^ Chi square test, *H* H for Kruskal *Wallis test*, Pairwise comparison bet. Each 2 groups was done using Post Hoc Test (Dunn's for multiple comparisons test). *p* p value for comparing between the three studied subtypes, *p*_*1*_ p value for comparing between Subtype 1 and Subtype 2, *p*_*2*_ p value for comparing between Subtype 1 and Subtype 3, *p*_*3*_ p value for comparing between Subtype 2 and Subtype 3 *: Statistically significant at p ≤ 0.05

Subtype 3 exhibited the highest levels of total cholesterol (TC: 220.50 ± 45.20 mg/dL), low-density lipoprotein (LDL: 140.30 ± 35.10 mg/dL), and triglycerides (TG: 180.60 ± 50.30 mg/dL). The 10-year ASCVD risk score was significantly elevated in Subtype 3 (mean 0.112 ± 0.108) compared to Subtype 1 (0.027 ± 0.030) and Subtype 2 (0.052 ± 0.060; p < 0.001). Notably, 17.4% of Subtype 3 patients were categorized as high-risk (ASCVD ≥ 20%) compared to only 2.7% in Subtype 1 and 5.6% in Subtype 2 (p < 0.001). Liver fibrosis markers were also significantly worse in Subtype 3 (FIB-4: 1.40 ± 0.78; NFS: 0.01 ± 1.02) (Table 2).

**Table 2:** Metabolic, lipid, and cardiovascular risk profiles by MASLD subtype

Parameter	Subtype 1 (n = 110)	Subtype 2 (n = 161)	Subtype 3 (n = 86)	P-value
Total cholesterol (mg/dL)	190.2 ± 40.1	200.30 ± 42.50	220.50 ± 45.20	< 0.001
LDL (mg/dL)	115.4 ± 30.2	125.60 ± 32.50	140.3 ± 35.10	< 0.001
HDL (mg/dL)	45.3 ± 11.1	43.50 ± 10.80	40.10 ± 10.20	0.008
Triglycerides (mg/dL)	140.5 ± 45.1	155.20 ± 47.80	180.60 ± 50.30	< 0.001
HbA1c (%)	5.8 ± 0.9	6.50 ± 1.20	7.80 ± 1.50	< 0.001
ASCVD risk score	0.027 ± 0.03	0.052 ± 0.060	0.112 ± 0.108	< 0.001
High ASCVD risk (≥ 20%), n (%)	3 (2.7)	9 (5.6)	15 (17.4)	< 0.001
FIB-4 score	0.95 ± 0.50	1.10 ± 0.60	1.40 ± 0.78	0.004
NFS score	−0.85 ± 0.90	−0.40 ± 0.95	0.01 ± 1.02	< 0.001

Values are presented as mean ± SD or n (%). *LDL* low-density lipoprotein,* HDL* high-density lipoprotein, *HbA1c* glycosylated hemoglobin, *ASCVD* Atherosclerotic Cardiovascular Disease, *FIB-4* Fibrosis-4, *NFS* NAFLD Fibrosis Score. P-values derived from ANOVA for continuous variables and Chi-square test for categorical variables

**Fig. 3:**
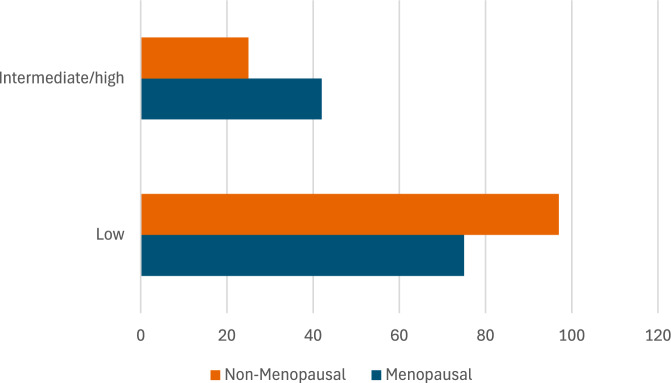
Menopausal females and CVR

**Fig. 4:**
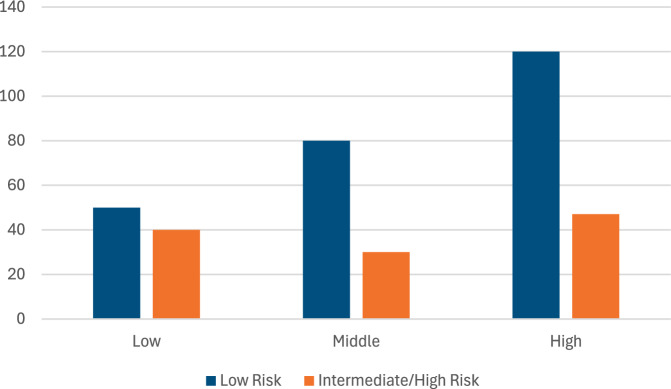
Physical activity and CVR

Patients with intermediate/high risk were more likely to be men (63.4% vs. 24.0%, p < 0.001), older (56.50 ± 9.59 vs. 44.69 ± 9.59 years, p < 0.001), smokers were (44.5% vs 55.5%, p < 0.001), hypertensive (39.0% vs. 12.0%, p < 0.001), and diabetics (73.2% vs. 27.3%, p < 0.001). Metabolic parameters, including LDL (135.20 ± 34.50 vs. 118.30 ± 31.20 mg/dL, p < 0.001), triglycerides (170.40 ± 49.50 vs. 145.60 ± 46.20 mg/dL, p < 0.001), and waist-to-hip ratio (0.95 ± 0.10 vs. 0.90 ± 0.08, p = 0.002), were significantly higher in the intermediate/high-risk group (Table 3). Menopausal females are significantly more likely to fall into intermediate/high-risk categories than non-menopausal females (Fig. 3). Physical activity (PA) level showed a borderline significant association with ASCVD risk (χ^2^ = 5.84, p = 0.054). Low-PA patients had 1.6 × higher odds of elevated risk compared to High-PA patients (Fig. 4).

**Table 3:** Factors associated with intermediate/high cardiovascular risk in MASLD patients

Factor	Low risk (< 7.5%) (n = 250)	Intermediate/high risk (≥ 7.5%) (n = 107)	P-value
Age (years)	44.69 ± 9.59	56.50 ± 9.59	< 0.001
Men, n (%)	60 (24.0)	68 (63.4)	< 0.001
Smoking, n (%)	20 (44.5%)	25 (55.5%)	< 0.001
Hypertension, n (%)	30 (12.0)	42 (39.0)	< 0.001
Diabetes, n (%)	68 (27.3)	78 (73.2)	< 0.001
LDL (mg/dL)	118.30 ± 31.20	135.20 ± 34.50	< 0.001
Triglycerides (mg/dL)	145.60 ± 46.20	170.40 ± 49.50	< 0.001
Waist-to-Hip ratio	0.90 ± 0.08	0.95 ± 0.10	0.002
FIB-4 score	1.00 ± 0.55	1.25 ± 0.70	0.015

Values are presented as mean ± SD or n (%). *LDL* low-density lipoprotein, *FIB-4* Fibrosis-4. P-values derived from Mann–Whitney U test for continuous variables and Chi-square test for categorical variables

In Multivariate logistic regression analysis identified Subtype 3 was a significant predictor (OR = 7.812, 95% CI 1.980–30.819, p = 0.003). Men (OR = 33.549, 95% CI 11.814–95.269, p < 0.001) and older age (OR = 1.171, 95% CI 1.116–1.229, p < 0.001) were also strong predictors (Table 4).

**Table 4:** Multivariate logistic regression analysis for predictors of intermediate/high ASCVD risk

	Univariate		^#^Multivariate	
	P	OR (LL – UL 95%C.I)	p	OR (LL – UL 95%C.I)
Subtypes				
Subtype 1		1.000		1.000
Subtype 2	0.299	1.444 (0.722 – 2.887)	0.154	2.186 (0.745 – 6.413)
Subtype 3	< 0.001^*^	5.963 (2.953 – 12.041)	0.003^*^	7.812 (1.980 – 30.819)
Men	< 0.001^*^	5.489 (3.238 – 9.304)	< 0.001^*^	57.203 (16.558 – 197.616)
Age (years)	< 0.001^*^	1.141 (1.103 – 1.180)	< 0.001^*^	1.188 (1.122 – 1.257)
Presence of HTN	< 0.001^*^	4.693 (2.644 – 8.330)	< 0.001^*^	9.044 (3.040 – 26.910)
Presence of DM	< 0.001^*^	7.273 (4.171 – 12.681)	< 0.001^*^	19.583 (4.152 – 92.377)
BMI (kg/m^2^)	0.463	1.015 (0.975 – 1.057)		
AST	0.946	1.000 (0.988 – 1.013)		
ALT	0.082	0.988 (0.974 – 1.002)		
GGT	0.781	1.001 (0.993 – 1.009)		
INR	0.285	0.101 (0.001 – 6.800)		
Platelet	0.208	0.997 (0.992 – 1.002)		
T. cholesterol	0.001^*^	1.007 (1.003 – 1.011)	0.001^*^	**0.952 (0.924 – 0.981)**
HDL	0.114	0.989 (0.976 – 1.003)		
LDL	0.003^*^	1.006 (1.002 – 1.010)	0.001^*^	1.012 (1.005 – 1.019)
TG	0.001^*^	1.005 (1.002 – 1.009)	0.036^*^	1.006 (1.000 – 1.011)
HbA1C	< 0.001^*^	1.331 (1.139 – 1.556)	0.369	.883 (0.673 – 1.159)
Waist circumference	0.032^*^	1.022 (1.002 – 1.042)	0.832	0.996 (0.957 – 1.036)
Waist/hip^$^	0.001^*^	1.874 (1.290 – 2.723)	0.016^*^	2.166 (1.158 – 4.052)
HOMA-IR	0.427	1.040 (0.944 – 1.146)		
QUIKI^$^	0.165	1.602 (0.824 – 3.115)		
APRI (> 1.5)	0.594	1.454 (0.367 – 5.754)		
ALBI	0.093	2.090 (0.884 – 4.943)		
FIB-4 (> 2.67)	0.013^*^	3.267 (1.280 – 8.339)	0.496	1.824 (0.324 – 10.280)
NFS (> 0.676)	< 0.001^*^	4.306 (2.136 – 8.681)	0.140	2.422 (0.748 – 7.836)
FLI (> 96)	0.034^*^	1.816 (1.048 – 3.147)	0.441	0.641 (0.207 – 1.987)
HSI(> 53)	0.402	0.754 (0.389 – 1.461)		

*HTN* hypertension, *DM* diabetes mellitus, *BMI* body mass index, *AST* aspartate aminotransferase, *ALT* alanine aminotransferase, *GGT* gamma-glutamyl transferase, *INR* international normalized ratio, *HDL* high-density lipoprotein, *LDL* low-density lipoprotein, *TG* triglycerides, *HbA1C* haemoglobin A1c, *WC* waist circumference, *W/H* waist-to-hip ratio, *HOMA-IR* homeostasis model assessment of insulin resistance, *QUIKI* quantitative insulin sensitivity check index, *APRI* AST-to-platelet ratio index, *ALBI* albumin-bilirubin index, *FIB-4* fibrosis-4 score, *HIS* hepatic steatosis index, *FLI* fatty liver index, *NFS* *NAFLD* fibrosis score, *M* mean, SD standard deviation, *OR* odds ratio, *CI* confidence interval Hosmer and Lemeshow Test (χ^2^ = 51.968^*^; p < 0.001^*^), *OR* Odd`s ratio, *C.I* Confidence interval, *LL* Lower limit, *UL* Upper Limit, #: All variables with p < 0.05 was included in the multivariate,*: Statistically significant at p ≤ 0.05, $: for each 0.1

## Discussion

This study illuminates the profound influence of metabolic burden on cardiovascular risk in patients with MASLD, offering a nuanced framework for risk stratification. By delineating three subtypes based on the constellation of metabolic criteria—diabetes, obesity, and metabolic dysregulation—we uncover a graded escalation in cardiovascular risk, with Subtype 3 patients, bearing the heaviest metabolic load, exhibiting the most severe cardiometabolic derangements.

Subtype 3 patients, characterized by diabetes, obesity, and multiple metabolic abnormalities, displayed markedly elevated LDL cholesterol, triglycerides, HbA1c, and ASCVD scores, alongside advanced liver fibrosis indices (FIB-4 and NFS). These findings resonate with prior evidence positioning MASLD as a dominant driver of cardiovascular morbidity, often eclipsing liver-related sequelae in this population [12–16]. The pronounced risk in Subtype 3 underscores the imperative for heightened clinical vigilance in this high-risk cohort.

Diabetes and hypertension, pivotal pillars of metabolic dysfunction, emerged as robust predictors of elevated cardiovascular risk. The near-ubiquitous presence of diabetes in Subtype 3, coupled with its independent association with intermediate to high ASCVD risk, corroborates the synergistic interplay between diabetes and hepatic steatosis in accelerating atherosclerosis [17, 18]. Hypertension and advancing age further amplify this risk, highlighting the necessity of early and comprehensive cardiometabolic screening in MASLD patients [19–21].

In addition to the metabolic burden, hormonal and lifestyle determinants may substantially influence cardiovascular and hepatic outcomes in MASLD. Our cohort exhibited a female predominance (66.9%), with mean ages ranging from 45.78 to 50.83 years across the subtypes—an age range that overlaps substantially with the menopausal transition in women. Hormonal changes during perimenopause and postmenopausal, particularly declining estrogen levels, have been associated with adverse changes in body fat distribution, insulin resistance, lipid profile, and vascular function, all of which may abolish the cardioprotective advantage typically observed in premenopausal women, thereby amplifying cardiovascular risk in MASLD cohorts [22]. Recent population-based analyses further demonstrate that postmenopausal women with MASLD display a 2.4-fold greater risk of cardiovascular disease compared to their premenopausal counterparts, underscoring the pivotal role of hormonal milieu [23]. Intriguingly, the current study added a point to the proposed power of MASLD to erode the cardiovascular protection traditionally afforded to postmenopausal women.

Smoking represents an additional aggravating factor, accelerating atherosclerosis, potentiating insulin resistance, and synergistically worsening cardiovascular outcomes in patients with metabolic liver disease [24]. In contrast, physical activity exerts a protective effect: leisure-time exercise is associated with lower all-cause and cardiovascular mortality in MASLD, and greater physical capacity independently correlates with reduced liver damage and lower ASCVD risk [25].

The lack of association between traditional liver enzymes (AST, ALT, GGT) and cardiovascular risk, contrasted with the predictive power of metabolic parameters such as LDL, triglycerides, and waist-to-hip ratio, reinforces MASLD’s identity as a systemic metabolic disorder rather than a solely hepatic condition [26, 27]. This paradigm shift underscores the need to prioritize cardiometabolic health in MASLD management.

The strong association of men, older age, diabetes, and hypertension with cardiovascular risk calls for tailored screening strategies. Men patients and those with diabetes or hypertension should be prioritized for annual cardiovascular evaluations, including lipid profiling and blood pressure monitoring.

Emerging evidence highlights that MASLD is not confined to individuals with obesity but also manifests in lean populations, challenging conventional paradigms and underscoring the centrality of metabolic dysregulation as a driving force across the full spectrum of body mass indices [28]. Interestingly, despite the high prevalence of obesity across all three MASLD subtypes, particularly Subtype 1 (92.7%) and Subtype 3 (100%)—BMI was not identified as an independent predictor of intermediate or high ASCVD risk in the multivariate analysis. This finding challenges the conventional assumption that obesity alone is a primary driver of cardiovascular risk. Recent studies suggest that obesity per se may not uniformly increase cardiovascular risk unless accompanied by metabolic derangements such as insulin resistance, hypertension, or dyslipidemia [26–28]. Furthermore, BMI lacks the ability to distinguish between visceral and subcutaneous adiposity, the former being more closely linked to atherosclerosis and cardiometabolic complications [29, 30]. Therefore, comprehensive metabolic profiling—including glycemic control, blood pressure, lipid levels, and markers of hepatic fibrosis—provides more accurate risk stratification than BMI alone in MASLD populations. These findings emphasize the need to assess the metabolic quality rather than the quantity of adiposity when evaluating cardiovascular risk in patients with MASLD.

The stratification of MASLD patients by metabolic burden offers actionable insights for clinicians. Subtype 3 patients, with their elevated ASCVD and fibrosis scores, represent a high-risk phenotype requiring aggressive cardiovascular risk mitigation. Clinicians should integrate routine ASCVD risk assessment, using tools like the ACC/AHA pooled cohort equations, into MASLD care, particularly for Subtype 3 patients. Early identification of this group enables targeted interventions, such as intensive glycemic control, lipid-lowering therapies (e.g., statins), and antihypertensive management, to forestall cardiovascular events. Lifestyle modifications—encompassing weight loss, regular physical activity, and dietary optimization (e.g., Mediterranean diet)—are paramount, especially given the high prevalence of obesity in Subtype 3 [31]. These interventions not only address cardiovascular risk but may also mitigate liver fibrosis progression, as suggested by the elevated FIB-4 and NFS scores in this subtype. Integrating menopausal status, smoking cessation, and physical activity assessment into clinical practice may therefore refine individualized cardiovascular and hepatic risk prediction and guide targeted preventive strategies.

A multidisciplinary approach, uniting hepatologists, cardiologists, endocrinologists, and dietitians, is essential to deliver holistic care. For instance, Subtype 3 patients may benefit from referral to cardiology for advanced risk assessment (e.g., stress testing) or to nutritionists for personalized dietary plans. Such collaborative care can optimize outcomes, particularly in resource-constrained settings like Egypt, where MASLD prevalence is rising.

Despite its strengths, -the large, well-characterized cohort and the rigorous use of validated ASCVD scores, this study has limitations. The single-center design which may restrict generalizability. Notably, the cross-sectional design precludes causal inferences, capturing only associations at a single time point. Prospective, longitudinal multicenter studies involving community-based and primary care populations, with larger, ethnically and geographically diverse cohorts are essential to validate these findings, track cardiovascular event incidence, fibrosis progression, and evaluate intervention efficacy. Uncontrolled confounders, including medication use (e.g., statins, antihypertensives, antidiabetic agents), and dietary habits may have influenced the observed associations. For example, statins may lower LDL cholesterol, while sedentary lifestyles or calorie-dense diets could exacerbate metabolic dysfunction, independently affecting ASCVD risk. The absence of inflammatory markers (e.g., C-reactive protein) or imaging-based assessments (e.g., coronary artery calcium scoring, liver elastography) further limits comprehensive risk profiling. While our current stratification of MAFLD subtypes based on the cumulative count of diagnostic criteria successfully established a clinically relevant gradient of metabolic dysfunction and cardiovascular risk, future classifications would benefit from more refined models would utilize machine learning techniques and cluster analysis to move beyond simple counting toward identifying distinct pathophysiological endotypes. Future studies should address these gaps to refine risk stratification.

## Conclusion

Stratifying MASLD patients by metabolic burden effectively identifies those at highest cardiovascular risk. Subtype 3 patients, with the greatest number of metabolic abnormalities, exhibited significantly elevated ASCVD risk and liver fibrosis markers. Men, older age, menopause, diabetes, and hypertension emerged as independent predictors of increased risk. These findings support integrating cardiovascular screening into MASLD management, particularly for patients with advanced metabolic dysfunction, to enable earlier intervention and improve long-term outcomes.

## Data Availability

Data is available at the corresponding author upon request.

## Abbreviations

ALT: Alanine aminotransferase
ALBI: Albumin-bilirubin index
APRI: AST-to-platelet ratio index
ASCVD: Atherosclerotic cardiovascular disease
AST: Aspartate aminotransferase
BMI: Body mass index
CI: Confidence interval
DM: Diabetes mellitus
FIB-4: Fibrosis-4 score
FLI: Steatotic liver inde
GGT: Gamma-glutamyl transferase
HbA1c: Hemoglobin A1c
HDL: High-density lipoprotein
HIS: Hepatic steatosis index
HOMA-IR: Homeostasis model assessment of insulin resistance
HTN: Hypertension
INR: International normalized ratio
LDL: Low-density lipoprotein
M: Mean
MASLD: Metabolic associated Steatotic liver disease
NFS: NAFLD fibrosis score
OR: Odds ratio
QR: Interquartile range
QUIKI: Quantitative insulin sensitivity check index
SD: Standard deviation
TG: Triglycerides
WC: Waist circumference
W/H: Waist-to-hip ratio
